# Obstructive Sleep Apnea and Type 2 Diabetes: An Update

**DOI:** 10.3390/jcm14155574

**Published:** 2025-08-07

**Authors:** Sandro Gentile, Vincenzo Maria Monda, Giuseppina Guarino, Ersilia Satta, Maria Chiarello, Giuseppe Caccavale, Edi Mattera, Raffaele Marfella, Felice Strollo

**Affiliations:** 1 Department of Precision Medicine, Vanvitelli University of Naples, 80131 Naples, Italy; s.gentile1949@gmail.com (S.G.); giuseppina.guarino@unicampania.it (G.G.); edi.mattera@unicampania.it (E.M.); 2 Research Department, Nefrocenter Research Network, 80059 Torre del Greco, Italy; e.satta@consorzionefrocenter.it (E.S.); mariachiarello97@gmail.com (M.C.); giuseppecaccavale@outlook.com (G.C.); 3 Diabetes Unit ‘‘Santissima Annunziata’’ Hospital Cento, 44042 Ferrara, Italy; v.monda@ausl.fe.it; 4 Department of Advanced Medical and Surgical Sciences (DAMSS), Vanvitelli University of Naples, 80138 Naples, Italy; raffaele.marfella@unicampania.it; 5 Department of Endocrinology and Metabolic Diseases, IRCCS San Raffaele Pisana, 00163 Rome, Italy

**Keywords:** sleep disorders, obstructive sleep apnea, type 2 diabetes, cardiovascular risk, obesity

## Abstract

Obstructive sleep apnea (OSA) syndrome is a severe, debilitating, and pervasive sleep disorder. OSA mainly affects people with obesity, type 2 diabetes mellitus (T2DM), hypertension, and dyslipidemia and is strongly associated with cardiovascular complications. Based on the bidirectional relationship between T2DM and OSA, the latter represents a risk factor for the former, and, vice versa, people with T2DM have a high risk of OSA. Mechanical and hormonal factors, inflammatory mediators, and a dysregulated autonomic nervous system contribute to the mechanisms underlying the disease. Treatment of OSA is necessary even if the available remedies are not always effective. In addition to traditional treatments, including lifestyle adaptations and bariatric surgery, CPAP equipment, i.e., a breathing device ensuring continuous positive pressure to keep the airways open during sleep, represents the most common treatment tool. More recently, pharmacological research has paved the way to newer seemingly effective therapeutic strategies involving, in particular, two hypoglycemic agent classes, i.e., sodium–glucose co-transporter 2 inhibitors (SGLT2-is) and glucagon-like peptide-1 (GLP-1) receptor agonists (GLP1-ras). This narrative review provides an update on all of the above.

## 1. Introduction

Obstructive sleep apnea (OSA) is a potentially debilitating, chronic respiratory disorder. It involves recurrent severe collapse of the upper airways, which severely impairs oxygen supply to all bodily tissues, thus causing repeated interruptions of night-time sleep. Sleep debt, in turn, precipitates daytime sleepiness, irritability, aggressiveness, and loss of regulatory control of hunger with consequent hyperphagia [1].

Its prevalence in the symptomatic form spans from 2 to 4% in the general population at any age [2,3]. Sleep fragmentation and intermittent hypoxia result in sympathetic activation, systemic inflammation, and oxidative stress, which, all together, significantly increase cardiometabolic risk, i.e., the development of type 2 diabetes mellitus (T2DM) independently of age and body mass index with related high morbidity and mortality [4,5]. Limited data is available, instead, on the pathophysiology and cardiometabolic impact of OSA in children with type 1 diabetes mellitus (T1DM) [6,7,8].

Indeed, several epidemiologic and experimental studies have described increased glucose levels and insulin resistance in patients with OSA [9,10,11]. According to Sleep Action for Health in Diabetes (AHEAD), 30% of obese people with T2DM suffer from OSA, thus accounting for several times higher prevalence than the general population [12,13].

The Sleep Heart Health Study, enrolling 2000 patients, pointed to a 1.44 odds ratio for abnormal glucose tolerance among patients with an Apnea Hypopnea Index (AHI) ≥ 15 compared to people with lower AHI levels. It also confirmed findings from the Nurses’ Health Study suggesting the development or exacerbation of T2DM as a consequence of curtailed sleep duration due to OAA-associated sleep fragmentation [14,15,16]. On the other hand, compared with those sleeping 7–8 h per night, individuals with ≤5 or <6 h sleep per night displayed adjusted odds ratios for T2DM of 2.51 (95% CI, 1.57–4.02) and 1.66 (95% CI, 1.15–2.39), respectively.

The purpose of this narrative review is to provide an update on obstructive sleep apnea, focusing on risk factors and clinical implications from a practical perspective, with a primary aim of benefiting clinicians. For this purpose and to facilitate reading, we divided the text schematically into numbered paragraphs, each dedicated to a relevant aspect.

**Figure 1:**
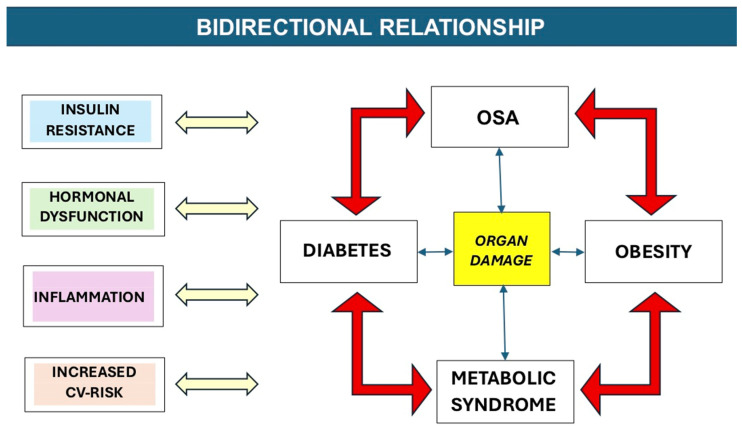
Graph schematically representing the bidirectional relationships among OSA, diabetes, obesity, metabolic syndrome, and resulting organ damage, with an eye to insulin resistance, hormonal dysfunction, inflammation, and increased cardiovascular risk considered simultaneously as causal factors and consequences of pathological aggregations.

Indeed, Figure 1 depicts the entire conceptual flow behind the text to lead the reader through the intricated relationship among the different factors contributing to the OSA–diabetes combination.

## 2. Insulin Resistance and β-Cell Dysfunction in OSA

Different animal and in vitro models have provided evidence of insulin resistance and β-cell dysfunction in OSA. In mice, intermittent hypoxia (IH) decreases insulin sensitivity measured via the glucose tolerance test (GTT) and homeostatic model assessment (HOMA) index [17,18,19]. IH hampers pancreatic β-cell proliferation and apoptosis and impairs the conversion of proinsulin to insulin in mice [20,21,22]. It also affects hepatocytes by increasing cellular glycogen content and gluconeogenic enzymatic activity and, when prolonged enough, proinflammatory signals like the nuclear transcription factor Kappa B (NFκB) and cytokines, i.e., interleukin-1β, interleukin-6, and macrophage inflammatory protein 2 [23]. Moreover, in adipocytes, IH increases levels of the proinflammatory cytokine resistin and downregulates adiponectin, an insulin-sensitizing hormone [24,25]. Finally, IH associates with sympathetic activation in both animal models and humans [26,27], as witnessed by results obtained in healthy awake adults undergoing intravenous GTT under control and 5 h IH conditions aimed at simulating moderate OSA, who also displayed decreased insulin sensitivity and glucose effectiveness despite unchanged pancreatic insulin secretion and serum cortisol levels [28].

Hypoxia-induced sleep fragmentation (SF) also affects glucose homeostasis per se, as shown in rodents by enhanced adiposity, insulin resistance, and hyperglycemia via increased levels of cortisol and inflammation or oxidative stress biomarkers. Also, experimentally induced SF with reduced slow-wave (i.e., deep) sleep duration impairs insulin sensitivity [29,30] in healthy humans and is associated with insulin resistance independent of age and obesity when combined with IH [31], while, in people with OSA or T2DM, is negatively affects glucose homeostasis [32,33,34,35,36].

## 3. Epidemiology

All of the abovementioned phenomena explain why, based on quantitative and validated measures, the prevalence of polysomnographically diagnosed OSA is higher in people with prediabetes and T2DM assessed based on fasting glucose or the GTT than in the general population, ranging from 18% in primary care [24] to 58% in an older cohort [25] and even 86% in obese T2DM patients [26]. In addition, T2DM seems to increase the likelihood of OSA [25] through autonomic and central nervous system dysfunction.

## 4. OSA and Metabolic Disease

Several studies have shown that OSA is associated with impaired glucose tolerance independent of obesity [37,38,39,40], the risk increasing in parallel with the severity of nocturnal hypoxia [40], as also witnessed in a longitudinal study of men without diabetes showing that OSA was an independent predictor of the development of insulin resistance [41] and others from North America, Europe, and Australia pointing to an increased risk of incident diabetes in moderate-to-severe OSA [42,43,44,45]. Despite the literature showing conflicting results after adjusting for possible confounders, including age, sex, and body mass index (BMI), a meta-analysis dating back to 2013 already supported these findings by estimating a 63% increased risk of incident DM in patients with OSA [46] and subsequent studies have suggested that OSA occurring during rapid eye movement (REM) sleep, when, typically, more severe oxygen desaturation events occur, results in insulin resistance and poorer glucose control [47,48].

## 5. Hormonal Involvement

OSA is strongly associated with obesity and related inflammation; an increasing number of investigators in the field have shown interest in adipose tissue biology and secretory patterns. Adipose tissue-related hormones endowed with a leading role are ghrelin, a 28-amino acid peptide hormone released by the stomach in the fasting state and having an appetite-stimulating and energy balance-controlling effect [49], and leptin and resistin, i.e., two powerful pro-inflammatory cytokines secreted by the adipose tissue and, therefore, classified as “adipokines”. The former has the primary role of controlling appetite [50]; the other is a cysteine-rich secretory protein typically endowed with insulin resistance-inducing properties [51]. Conversely, the most abundant circulating adipokine, called adiponectin, reduces the production and activity of pro-inflammatory cytokines [52]. Human obesity is associated with increased leptin and resistin levels, as well as decreased adiponectin and ghrelin levels [49,53,54,55], while significant relationships have been shown among obesity, adipokines, systemic inflammation, and atherosclerosis [56]

Indeed, compared to fifteen age-matched controls, fifty-five newly diagnosed OSA patients displayed significantly higher ghrelin levels (*p* < 0.05) positively correlating with the AHI (r = 0.237, *p* < 0.05) and Epworth sleepiness scale (ESS) (r = 0.28, *p* < 0.05) in the absence of any differences in circulating leptin, adiponectin, and resistin concentrations, which also failed to correlate with any polysomnographic parameters [57].

When turning to non-adipose-tissue-related hormones, we found that obese people with OSA and, even more, nonobese men and slightly obese women with OSA have increased cortisol levels, significantly decreasing after short-term continuous positive airway pressure (CPAP), thus pointing to a possible protective CPAP effect against hypertension and other comorbidities [58,59], even including osteoporosis [60]., frequently associated with chronic HPA axis activation.

Comprehensively, some components of the hormonal milieu influence at least in part the expression of OSA.

## 6. OSA and Inflammation

As already mentioned, IH, SF, enhanced sympathetic system activity, and disrupted homeostasis are associated with systemic low-grade inflammation (LGI) [61]. As activated inflammation pathways can either be a cause or consequence of OSA [62], understanding the causal relationship between CRP and TNF-α, known as the key systemic inflammation protein markers, and OSA helps go deeper into the underlying mechanisms and develop effective treatment strategies against OSA. Indeed, the Apnea Hypopnea Index (AHI) and blood oxygen desaturation in OSA are associated with CRP levels [63,64,65] and, with only a few exceptions [66], epidemiological OSA investigations and a meta-analysis including 15 studies showed elevated CRP and TNF-α levels [67,68,69,70]. In particular, cellular and biopsy-based experiments have suggested that OSA promotes vascular inflammation through increased CRP and TNF-α levels, and a study based on sophisticated techniques found a positive association between CRP and TNF-α and OSA severity, together with a milder CPAP protective effect on CRP than TNF-α [70,71,72]. Such findings might depend on the presence of obesity, arterial hypertension, and diabetes in samples generally chosen for this kind of investigation [73,74,75], as an RCT excluding these comorbidities reported a significant CRP reduction [76,77,78]. Indeed, many confounding factors limit our understanding of the association between inflammation and OSA. For instance, besides being the most common risk factor for OSA [79], obesity has been shown to increase the levels of cytokines, including CRP and TNF-α [80]. Recently, OSA has been shown to alter the protein composition, including CRP, of serum extracellular micro-vesicles, which may contribute to its complications [81]. Also, Gaines et al. reported that CRP is the mediator between obesity and OSA, indicating that the release of CRP by visceral adipocytes plays a causative role in the development of OSA [82].

Therefore, when putting together the evidence accumulated so far in the field, despite the need for further investigation, especially in the field of LGI-dependent neurocognitive defects, CRP stands out as the inflammation marker more remarkably linked to OSA through a mild vicious cycle.

## 7. Effects of OSA on Type 2 Diabetes

### 7.1. Metabolic Control

Several cross-sectional studies have found a detrimental impact of OSA on glycemic control in type 2 diabetes [48,83,84,85]. According to Aronsohn et al. [84], who studied 60 people with type 2 diabetes, polysomnography-derived AHI positively correlated with HbA1c after controlling for multiple confounders, and the effect size of the AHI on HbA1c was more relevant than that of some hypoglycemic drugs. However, possibly because of the high prevalence of obese, older participants with a longer duration of diabetes, the investigators of a subanalysis of a large prospective study involving 305 of 5145 participants from 4 of 16 centers failed to confirm such a correlation and only found some between fasting glucose and sleep efficiency [86]. Also, despite showing that HbA1c could predict AHI in glucose-intolerant patients, others found a stronger inverse correlation between oxygen saturation and HbA1c in people with T2DM, thus pointing to a somewhat elusive relationship between the two parameters when failing to control for all possible confounding factors [87].

Taken together, all of the above points to the fact that, in T2DM, moderate-to-severe OSA is associated with poor metabolic control.

### 7.2. Organ System Dysfunction Associated with T2DM

OSA also aggravates cardiovascular disease (CVD) through increased sympathetic drive and enhanced oxidative stress, which later impairs endothelial nitric oxide-dependent vasodilation [88] and precipitates a pro-inflammatory and hypercoagulable state [89]. Indeed, the significant association between AHI-related OSA severity and stroke risk (OR 2.5) found in old, obese subjects with T2DM [90] warrants further investigation in lean and younger subjects with T2DM before accepting and generalizing any conclusions in this regard.

The literature also empirically suggests that OSA promotes chronic kidney disease progression in individuals with and without T2DM [91] and, as reported in 500 Japanese patients with T2DM, increases the risk of hypertension and hyperlipidemia with micro- and macro-albuminuria [92]. A British study led to analogous results independently of obesity [93], and a recent systematic review and meta-analysis found OSA severity to be associated with a 1.73-fold increase in the risk of diabetic kidney disease [94].

A cross-sectional study involving over 200 hospitalized T2DM patients showed peripheral neuropathy, and especially its severe expression, to be more prevalent in those with OSA, who also displayed high levels of nitrotyrosine and lipid peroxides associated with nocturnal hypoxemia, thus suggesting oxidative stress as a possible underlying mechanism for peripheral neuropathy [95].

Different cross-sectional studies have also pointed to an increased prevalence and severity of retinopathy and maculopathy in people with T2DM, with an increased incidence of ocular complications in patients with OSA [96,97,98].

All of the above indicates that OSA is a further risk factor for any signs of organ system dysfunction in T2DM.

### 7.3. Effects of OSA Treatment on Diabetes

OSA treatment invariably includes better sleep habits and improved lifestyle, with weight loss attained through reduced caloric intake and increased physical activity. Indeed, OSA becomes less severe after weight loss, whether medically or surgically attained, but it is not clear whether the better metabolic control observed in T2DM patients depends on OSA amelioration independently of weight loss itself [99,100].

As pointed out later, ear, nose, and throat or maxillofacial surgery is also used against OSA when needed, and, recently, maxillary advancement devices fitted by orthodontists have become increasingly popular, but the effects of the latter in T2DM are still unclear. However, the low rate of OSA remission after bariatric surgery suggests that OSA does not depend only on obesity per se but also on individual jaw anatomy [101].

Continuous positive airway pressure (CPAP) used during night sleep is the first line and most effective treatment for OSA, which has also been proven to positively affect glucose metabolism in obese and nonobese subjects with prediabetes by improving insulin sensitivity and the 24 h blood pressure profile compared to a placebo [102,103], as also shown by a meta-analysis of randomized, controlled trials with a total of ∼240 subjects free of T2DM showing a significantly improved HOMA index after CPAP treatment [104]. Indeed, several individual studies and meta-analyses from the past 5 years have shown a clear positive influence of CPAP treatment on metabolic markers more evidently in prediabetes than in T2DM [103,104,105,106,107,108,109,110,111,112]. The latter finding probably depends on the fact that T2DM reflects a more advanced form of metabolic dysregulation, more likely affecting older patients with comorbidities. The abovementioned observation holds especially true in the case of high adherence to CPAP treatment for at least 3 months, moderate-to-severe OSA, obesity, and poorly controlled T2DM [103,106,107,108,109,110,111,112,113,114], despite a lower effect size than that of weight loss and oral hypoglycemic agents [113].

All of these findings point to the need for early OSA diagnosis and sustained treatment adherence in those at risk of T2DM.

## 8. Effects of Diabetes Treatment on OSA

Reducing glycemic variability and HbA1c levels improves OSA severity and is therefore necessary in T2DM [115,116]. In this regard, now that innovative antihyperglycemic, non-hypoglycemic drugs, including DPP-4i, GLP1-ra, and SGLT-2is, have come onto the market, how to attain better metabolic control is also relevant.

### 8.1. Glucagon-like Peptide-1 (GLP-1) Receptor Agonists (GLP-1ras)

For their durable appetite and glucagon-suppressing effects on weight loss and glycemic control, this class of drugs has attained a significant therapeutic role in people with obesity and T2DM. Indeed, by being effective against endothelial dysfunction-related diabetes complications and most OSA-related cardiovascular complications, including hypertension, arrhythmias, heart failure, acute myocardial infarction, and even sudden cardiac death, pharmaceutical companies have invested considerable efforts in this field [117]. Conversely, to the best of our knowledge, the interest in developing new research in the field of CPAP has not increased in parallel. Indeed, clinical studies comparing CPAP and pharmacotherapy would be desirable.

Based on the results of Jiang et al. in 90 patients with T2DM and CPAP-treated OSA, 3 months were enough for liraglutide, i.e., a widely used GLP1-ra, to improve minimum oxygen saturation and reduce AHI, BMI, and mean systolic blood pressure (SBP) without any clinically relevant side effects [118].

Also, a randomized, double-blind trial involving CPAP-refusing, obese non-diabetic participants with moderate or severe OSA undergoing 3.0 mg liraglutide treatment for 32 weeks plus diet and exercise showed a greater decrease in AHI, body weight, HbA1c and SBP with liraglutide than with a placebo and a significant association between the degree of weight loss and OSA improvement [119].

More recently, tirzepatide, i.e., a dual receptor co-agonist of GLP-1 and GIP (gastric inhibitory polypeptide/glucose-dependent insulinotropic polypeptide) intended as an anti-hyperglycemic drug, was used for 52 weeks, with and without CPAP, in two phase 3, double-blind, randomized, controlled trials involving adults suffering from moderate-to-severe OSA and obesity. AHI, body weight, hypoxic burden (i.e., patient-reported sleep impairment and disturbance), high-sensitivity C-reactive protein concentrations, and SBP significantly decreased with tirzepatide [120].

### 8.2. Dipeptidyl Peptidase-4 Inhibitors (DPP-4is) or “Gliptins”

Thanks to their significant glucagon-suppressive anti-hyperglycemic effect, added to neutral effects on BMI, oral DPP-4is, i.e., a user-friendly class of “incretin-mimetic drugs” exploiting glucagon-like peptide-1 (GLP-1) potential, have been increasingly utilized in T2DM patients inadequately controlled with insulin therapy during the last two decades. However, they have not been extensively investigated in patients with OSA, despite the lack in such cases of the typical positive association between BMI and DPP-4, i.e., the GLP-1-degrading enzyme against which the abovementioned drugs are directed. Indeed, the results from 96 non-diabetic subjects with OSA showed elevated fasting GLP-1 without parallelly increased DPP-4 levels, eventually influencing fasting glucose levels [121]. In our opinion, such an observation warrants further investigation.

### 8.3. Sodium–Glucose Co-Transporter 2 Inhibitors (SGLT2-Is) of “Gliflozins”

The above are the newest anti-hyperglycemics to appear on the diabetes-related market. They have literally revolutionized T2DM treatment and promise to become a powerful tool against OSA as well through the following mechanisms [122]: (i) sympathetic activity inhibition [123,124], counteracting cardiac autonomic neuropathy (CAN), cardiovascular defects, and renal dysfunction [125]; (ii) direct benefits on renal function [126,127] preventing CAN from entering a renal dysfunction-related vitious circle [128,129]; and (iii) increased Ht expected to reduce nocturnal hypoxia [130,131,132,133].

Indeed, studies on patients with T2DM patients with OSA showed SGLT-2is to almost halve the AHI (31.9 ± 18.0 to 18.8 ± 11.5 events/h; *p* = 0.003) while reducing HbA1c and BMI without affecting BP [134,135]. Besides counteracting cardiovascular and renal dysfunction regardless of OSA, empagliflozin proved to lessen the OSA risk [136] by mitigating leptin hyperproduction and hyperactivity [137,138] and increasing adiponectin levels [138]. By significantly reducing glucose levels, BMI, blood pressure, and AHI and improving night-time hypoxemia and excessive daytime sleepiness, dapagliflozin seems to be a promising tool against OSA as well [135].

In addition, SGLT2is enhance lipolysis and fatty acid beta-oxidation and thus may prevent OSA by decreasing the amount of visceral and subcutaneous adipose tissue. Moreover, based on experimental and clinical evidence, they may avoid the onset and progression of NAFLD, whose prevalence exceeds 50%, and thus exert further protective effects against heart failure with preserved ejection fraction in people with T2DM and OSA [139].

According to the results of a recent paper on patients with T2DM and OSA, 6 months of treatment with tofoglifozin, i.e., a newcomer in the class apparently unable to ameliorate cardiorenal function, also reduced AHI together with HbA1c, body weight, and water content; as for the linear relationship observed between the changes in body water content and AHI (r = 0.642, *p* = 0.045), the positive effect might be interpreted as depending on decreased body water content [140].

## 9. Surgery

Snoring is a common, disturbing symptom observed in persons with obesity of either sex, especially with advancing age. Although appearing benign, snoring is annoying and may have detrimental effects on health, thus warranting further investigation, particularly in the context of temporomandibular misalignment. However, unfortunately, it may represent a pre-OSA stage. Indeed, the noise may progressively increase in volume and alternate with silent shortness-of-breath phases, causing sudden, noisy, choking awakenings [141]. Such a change characterizes the shift from snoring to OSA.

Treatment of possible oropharyngeal causes involves the anatomical structures of the nose, palate, tongue, and jaw in a personalized way and based on careful planning of the best option. Indeed, the surgeon has to examine the history of the single subject (including, e.g., simple and non-invasive approaches such as the use of nasal patches, decongestants, and anti-allergic sprays), as well as a careful study of individual anatomical characteristics.

Surgical approaches can include removal of nasal turbinates and palatine and adenoid tonsils, pharyngoplasty, and surgical correction of the back of the tongue (even with modern techniques using robotic surgery). However, more complex operations are sometimes necessary, such as maxillomandibular advancement [142,143].

In greater detail, all types of intervention aim to minimize obstacles to the free flow of air during respiratory phases, particularly in the lying position, during which the soft tissues of the back of the mouth and pharynx tend to collapse.

Lateral pharyngoplasty, also called expansion sphincteroplasty, is often performed in patients with a narrow throat or webbing of the posterior arch of the palate. In the case of associated large tonsils, especially lingual tonsils, tonsillectomy is required.

In those with an elongated soft palate and uvula, a palatal z-plasty can shorten the palate and avoid vibrations while preventing soft palate narrowing during the healing process.

Tongue reduction surgery may also improve airflow and breathing in the case of an enlarged tongue. When the anatomical structure of the tongue causes significant obstruction, thyrohyoidopexy may prove helpful by repositioning the hyoid bone and pulling the tongue and epiglottis down and forward. Such a technique allows for recovery within a few days, significantly reducing the weeks commonly expected with other methods, which the surgeon can still exploit afterwards, if needed.

The genioglossus muscle, which attaches to the underside of the chin and moves the tongue forward during contraction, may also undergo surgical advancement in the case of moderate OSA. However, when OSA is more severe, maxillomandibular advancement (MMA) surgery may lead to a significant expansion of the airway and thus solve the problem more radically. However, caution is required when approaching surgery, considering that central sleep apnea or other systemic diseases are contraindications for it.

## 10. Conclusions

A credible estimate is that about 1 billion people have more than five obstructive AHI events per hour of sleep and that about 425 million people have moderate or severe such forms (>15 AHI events per hour of sleep) requiring specific treatments. Many patients also live with obesity, and many more with T2DM.

Considering that sleep debt significantly deteriorates quality of life and performance, it is necessary to strive against what can be seen as a sort of social plague. Indeed, the Italian National Health System recognizes a 30% disability (not full handicap) for those who suffer from OSA, but, quite often, comorbidities, including diabetes, hypertension, dyslipidemia, and cardiovascular diseases, are also present, which significantly increase the economic, social, and personal burden of the disease [144].

CPAP is still the treatment of choice, in addition to lifestyle changes, as bariatric surgery and pharmacological tools are still failing to ensure success for all possible patients. However, despite the number of dedicated studies still being limited, the abovementioned new drugs meant to treat diabetes and obesity can be viewed as frontiers in OSA prevention and therapy by addressing the pathogenic mechanisms underlying the disease while ensuring direct cardio- and nephro-protective effects, which further reduce the severity of OSA complications.

## Data Availability

Data sharing does not apply to this article as no datasets were generated or analyzed during the current study.
